# Endoscopic glue injection therapy with N-butyl-2-cyanoacrylate for bleeding from isolated large rectal varices

**DOI:** 10.1055/a-2378-6198

**Published:** 2024-08-16

**Authors:** Shigenaga Matsui, Hiroshi Kashida, Masahiro Takita, Masatoshi Kudo

**Affiliations:** 1 Gastroenterology and Hepatology, Kindai University Faculty of Medicine, Osaka-Sayama, Japan

A 64-year-old woman with history of hepatitis C cirrhosis (Child-Pugh class C) was admitted to our institution with hematochezia. Her esophagogastric varices had been treated at our hospital 6 and 3 years previously by balloon-occluded retrograde transvenous obliteration (B-RTO) and endoscopic injection sclerotherapy (EIS), respectively, and she was being treated for refractory ascites with concentrated ascites reinfusion therapy (CART). She was found to be anemic (hemoglobin 6.0 g/dL) and renal dysfunction was also noted, with a blood urea nitrogen level of 117 mg/dL and creatinine level of 2.87 mg/dL. Plain abdominal computed tomography (CT) revealed liver cirrhosis with massive ascites ( Fig. 1 ).

**Fig. 1:**
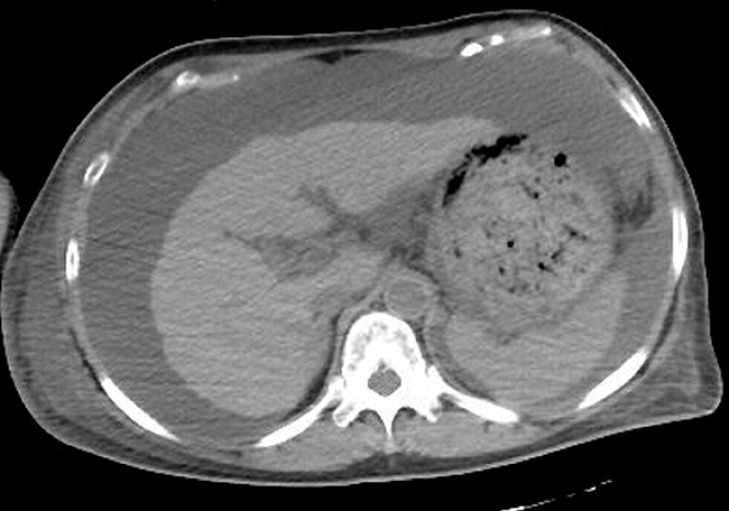
Plain abdominal computed tomography scan showing liver cirrhosis with massive ascites.

Colonoscopy revealed isolated large rectal varices with erosion, which were identified as the source of her bleeding ( Fig. 2 **a** ). To decrease the blood flow in the rectal varices, we first injected 50% glucose solution into the varices before performing endoscopic glue injection. We then immediately performed glue injection via three punctures into the rectal varices using a mixture of 1.5 mL N-butyl-2-cyanoacrylate (NBCA) and 0.5 mL Lipiodol ( Fig. 2 **b** ; Video 1 ). A computed tomography scan after the endoscopic glue injection therapy showed the injected NBCA occupying the rectal varices ( Fig. 3 ). There were no complications after endoscopic glue injection, but the patient died 5 months later of liver failure having had no further episodes of hematochezia.

**Fig. 2:**
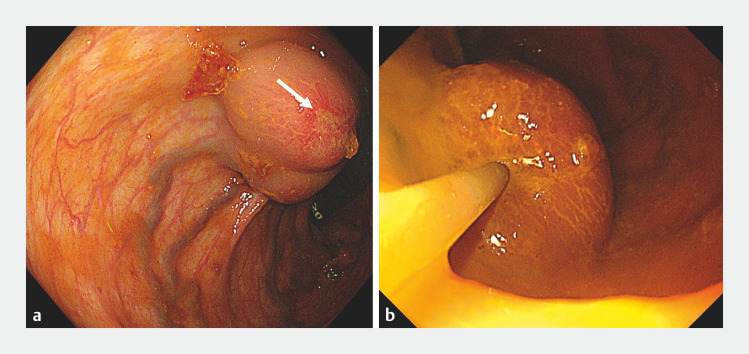
Endoscopic images showing: **a** bleeding isolated large rectal varices with erosion (arrow); **b** endoscopic glue injection being performed at the rectal varices.

**Fig. 3:**
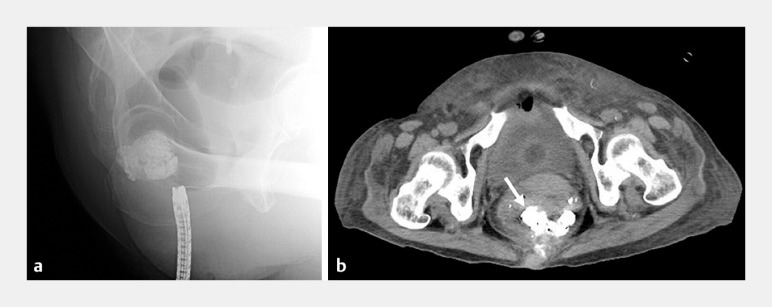
The injected cyanoacrylate is seen to be occupying the rectal varices after glue injection on: **a** fluoroscopic imaging; **b** computed tomography scanning (arrow).

Successful treatment for bleeding from isolated large rectal varices using endoscopic glue injection therapy with N-butyl-2-cyanoacrylate.Video 1

Management of rectal varices can be achieved by endoscopic band ligation (EBL), EIS, interventional radiology (IVR) such as B-RTO and transjugular intrahepatic portosystemic shunt (TIPS), and surgical management 1 . EBL carries a high risk of rebleeding for large isolated rectal varices, and IVR was not indicated in this case owing to the presence of chronic renal failure. Endoscopic glue injection therapy is the standard treatment for gastric varices 2 , it is also useful for rectal varices. In conclusion, endoscopic glue injection with NBCA is recommended for initial hemostasis of bleeding rectal varices, being quick, safe, and minimally invasive, in the presence of severe liver dysfunction and chronic renal failure.

Endoscopy_UCTN_Code_TTT_1AQ_2AZ

## Notes

Endoscopy E-Videos https://eref.thieme.de/e-videos
*E-Videos* is an open access online section of the journal *Endoscopy* , reporting on interesting cases and new techniques in gastroenterological endoscopy. All papers include a high-quality video and are published with a Creative Commons CC-BY license. Endoscopy E-Videos qualify for HINARI discounts and waivers and eligibility is automatically checked during the submission process. We grant 100% waivers to articles whose corresponding authors are based in Group A countries and 50% waivers to those who are based in Group B countries as classified by Research4Life (see: https://www.research4life.org/access/eligibility/ ). This section has its own submission website at https://mc.manuscriptcentral.com/e-videos .
